# Bisphenol A: vom Saal and Hughes Respond

**Published:** 2006-01

**Authors:** Frederick S. vom Saal, Claude Hughes

**Affiliations:** Division of Biological Sciences, University of Missouri, Columbia, Missouri, E-mail: vomsaalf@missouri.edu; Department of Biology, East Carolina University, Greenville, North Carolina

Our commentary describing the extensive new literature reporting low-dose effects of bisphenol A (BPA) in experimental animals (vom Saal and Hughes 2005) was written in response to a report from the Harvard Center for Risk Analysis (HCRA) by Gray et al. (2004), who concluded that “the weight of the evidence for low-dose effects [of BPA] is very weak.” The HCRA report was funded by the American Plastics Council and involved a selective review of only 19 of a much larger number of studies that could have been reviewed. In our commentary we showed that a comprehensive review of the now extensive literature concerning studies in experimental animals that used doses of BPA within the range of human exposure led to exactly the opposite conclusion from that reached in the HCRA report (Gray et al. 2004), which was released 2.5 years after it was written.

At this time there are only two published epidemiologic studies showing a relationship between blood levels of BPA and diseases in humans. In his letter, Politch focuses his attention on a single study by Takeuchi et al. (2004) that describes a relationship between BPA in blood and polycystic ovary disease (PCOS) in Japanese women. In a second recently published article, Sugiura-Ogasawara et al. (2005) reported a relationship between blood levels of BPA and recurrent miscarriage in Japanese women. Politch seeks to deflect attention from the central issue of our review by focusing only on the study by Takeuchi et al. (2004) and stating that such studies “cannot address causal relationships” and suggesting that “appropriately controlled” human studies are required. We are certain that readers of *Environmental Health Perspectives* (*EHP*) realize that these are criticisms that can be directed at all epidemiologic studies, which can never achieve the control required in laboratory experiments. Additionally, there is always some risk in arguing the methodologic details of a peer-reviewed publication in one field of scientific research (epidemiology) when the commentator’s core expertise (biopsychology) lies elsewhere. Most importantly, based on his criticism of the levels of BPA reported in the blood of women by Takeuchi et al. (2004), Politch appears to be unaware of the large literature concerning the levels of BPA in human blood, urine, and tissues from studies conducted in different regions of the world reporting virtually identical mean and/or median values. For example, in a recent study at the Centers for Disease Control and Prevention, Calafat et al. (2005) found BPA in 95% of the human urine samples they assayed—in the same range reported in human blood in other studies (e.g., Schonfelder et al. 2002; Tan and Mohd 2003). All of this published literature is listed in a document available on the University of Missouri Endocrine Disruptor web site (Endocrine Disruptors Group 2005).

One point-of-view expressed by Politch that we strongly support is the proposition that human studies linking developmental exposure with adult disease are also required, based on the extensive evidence that the developing fetus and neonate are the most vulnerable to endocrine disruption. We hope that the planned National Children’s Study will address this issue and begin to characterize which exposures are and are not consequential for human health. In the absence of such a study, which will take decades to complete, we rely on experimental studies in animals to make decisions regarding the potential hazards posed by chemicals.

Our comment that the epidemiologic evidence “adds to our concern” about the potential hazards posed to humans by BPA hardly qualifies as justification for the criticism that we “overstated the importance” of this or any other single study. Our concern about the potential hazards of BPA to humans is justified by the fact that the limited epidemiologic studies do follow and generally support findings from over 125 experiments with laboratory animals showing that low doses of BPA cause adverse effects on a wide range of outcomes. We also pointed out in our article (vom Saal and Hughes 2005) that 100% of the studies showing significant effects of BPA in laboratory animals were funded by government agencies, and 100% of the studies funded by chemical corporations conclude that the same low doses of BPA do not cause significant effects. What is crucial in relation to the critique by Politch is that the two epidemiologic studies relating BPA in blood to diseases in women are consistent with the findings from studies of the hazards of BPA in animals at doses that lead to blood levels in animals within and below those detected in human blood.
